# Multivitamin supplementation in HIV infected adults initiating antiretroviral therapy in Uganda: the protocol for a randomized double blinded placebo controlled efficacy trial

**DOI:** 10.1186/1471-2334-12-304

**Published:** 2012-11-15

**Authors:** David Guwatudde, Amara E Ezeamama, Danstan Bagenda, Rachel Kyeyune, Fred Wabwire-Mangen, Henry Wamani, Ferdinand Mugusi, Donna Spiegelman, Molin Wang, Yukari C Manabe, Wafaie W Fawzi

**Affiliations:** 1 School of Public Health, Makerere University College of Health Sciences, P.O. Box 7072, Kampala, Uganda; 2 Department of Nutrition, Harvard School of Public Health, Boston, MA, USA; 3 Infectious Diseases Institute, Makerere University College of Health Sciences, Kampala, Uganda; 4 Muhimbili University of Health and Allied Sciences, Dar-es-Salam, Tanzania; 5 Department of Epidemiology, Harvard School of Public Health, Boston, MA, USA; 6 Department of Medicine, Division of Infectious Diseases, John Hopkins University, Baltimore, MD, USA; 7 Department of Global Health and Population, Harvard School of Public Health, Boston, MA, USA

**Keywords:** HIV infected adults, HAART, Micronutrient supplementation, Nutrition, Randomized double-blind placebo-controlled trial, Trial protocol, Uganda, Sub-Saharan Africa

## Abstract

**Background:**

Use of multivitamin supplements during the pre-HAART era has been found to reduce viral load, enhance immune response, and generally improve clinical outcomes among HIV-infected adults. However, immune reconstitution is incomplete and significant mortality and opportunistic infections occur in spite of HAART. There is insufficient research information on whether multivitamin supplementation may be beneficial as adjunct therapy for HIV-infected individuals taking HAART. We propose to evaluate the efficacy of a single recommended daily allowance (RDA) of micronutrients (including vitamins B-complex, C, and E) in slowing disease progression among HIV-infected adults receiving HAART in Uganda.

**Methods/Design:**

We are using a randomized, double-blind, placebo-controlled trial study design. Eligible patients are HIV-positive adults aged at least 18 years, and are randomized to receive either a placebo; or multivitamins that include a single RDA of the following vitamins: 1.4 mg B1, 1.4 mg B2, 1.9 mg B6, 2.6 mcg B12, 18 mg niacin, 70 mg C, 10 mg E, and 0.4 mg folic acid. Participants are followed for up to 18 months with evaluations at baseline, 6, 12 and 18 months. The study is primarily powered to examine the effects on immune reconstitution, weight gain, and quality of life. In addition, we will examine the effects on other secondary outcomes including the risks of development of new or recurrent disease progression event, including all-cause mortality; ARV regimen change from first- to second-line therapy; and other adverse events as indicated by incident peripheral neuropathy, severe anemia, or diarrhea.

**Discussions:**

The conduct of this trial provides an opportunity to evaluate the potential benefits of this affordable adjunct therapy (multivitamin supplementation) among HIV-infected adults receiving HAART in a developing country setting.

**Trial registration:**

Clinical Trial Registration-URL: http://www.clinicaltrials.gov. Unique identifier: NCT01228578

## Background

Vitamins and minerals are important micronutrients for optimal immune system function [1]. Nutrient deficiencies and their effects on immune function, disease progression, and their markers of health have been documented in HIV-infected persons [2,3]. Although the widespread introduction of highly active antiretroviral therapy (HAART) in most developing countries has dramatically reduced morbidity and mortality among HIV-positive individuals, under-nutrition that is common in these settings, coupled with wasting that is characteristic of HIV infection, may affect optimal immunologic response after HAART initiation.

A number of studies conducted among HIV-infected adults during the pre-HAART era, have shown that multivitamin supplementation enhances immune reconstitution, reduces viral load, improves overall clinical outcomes, and reduces mortality [4-6]. Among such patients, multivitamins could slow disease progression and prolong the time before HAART needs to be initiated. But only a few interventional trials with small sample sizes have explored multivitamin supplementation in relation to HIV progression during HAART. It is unclear from these studies whether the micronutrient supplementation-associated health benefits observed during the pre-HAART era would also be observed among HIV-infected patients on HAART; with some of intervention studies suggesting beneficial treatment outcomes [7-11], whereas others did not show any beneficial treatment outcomes [12,13]. Sufficient data are therefore lacking to evaluate whether micronutrient supplementation during HAART has an impact on treatments outcomes. A daily dose of multivitamins costs less than 5 United States cents and adequate supply for the duration of a year would cost less than $15. Identifying whether such a low-cost intervention has an impact on HIV disease progression among individuals on ART is an important question to answer given the large potential for improving quality of life and the cost-savings that could result.

The primary aim of this trial is to determine whether one recommended dietary allowance (RDA) dose of oral multivitamin supplements (including vitamins B-complex, C, and E) given daily for 18 months will: (1) improve immune reconstitution (indicated by CD4 cell count); (2) improve weight gain, and (3) improve quality of life.

## Methods

### Study design

This is a randomized, double-blind, placebo-controlled trial. We are randomizing participants to receive daily oral supplements in one of two experimental groups: Group 1: multivitamins, including a single RDA of the following vitamins: 1.4 mg B1, 1.4 mg B2, 1.9 mg B6, 2.6 mcg B12, 18 mg niacin, 70 mg C, 10 mg E, and 0.4 mg folic acid; or Group 2: placebo which consists of an inactive pill of the same size, packaging and coloration as the active multivitamin tablets.

### Setting

The trial is being conducted at the Infectious Diseases Institute (IDI), Makerere University College of Health Sciences. IDI provides care and treatment for HIV infected persons as one of its core functions; over 10,000 people receive care through the IDI HIV care clinic, approximately 6,000 of which are on antiretroviral therapy (ART).

### Recruitment and enrollment

Prior to physical contact with any patient by the study staff, a designated clinical staff at the IDI HIV care clinic conducts chart reviews of prospective HIV-positive patients ahead of their scheduled clinic visit to determine their potential eligibility for HAART, or their duration of HAART use for those already on ARVs. This information is made available to the trial Study Coordinator, who uses the information to approach potential trial participants identified upon arrival at the IDI HIV care clinic for their next scheduled appointments. At this time, the trial clinical staff informs the patient about the trial and invites them for further eligibility screening and possible enrollment into the trial. Prior to enrollment, trial participation eligibility is documented, and written informed consent sought from eligible patients. Patients are provided with information on the objectives of the study and confidentiality issues are emphasized. At the end of this session, patients are given the opportunity to enroll in the trial at that time and are continued on the appropriate HIV care per Uganda’s standard of care guidelines. Those who want to discuss their participation in the trial with family members are given an opportunity to do so and may enroll at their next scheduled clinic visit. Eligible subjects must meet the following inclusion criteria: a) men or women aged >=18 years old, b) HIV-positive, c) initiating anti-retroviral therapy at the time of randomization or have been on HAART for no longer than 6 months, d) have no intention of migrating, or re-locating more than 20 km outside of the IDI within the next 18 months after enrollment, e) agree to allow home visit(s) and subsequent follow-up contacts as part of the study, and f) provide written informed consent.

Subjects with any of the following criteria are excluded: a) Because iron and folic acid supplementation are standard of care for pregnant women per the Ugandan Ministry of Health standards, women with a positive pregnancy test are excluded from participation in the trial as we would not have adequate statistical power to examine this factor as an effect modifier; b) Individuals who are very ill and unable to consent.

### Randomization

**Figure 1:**
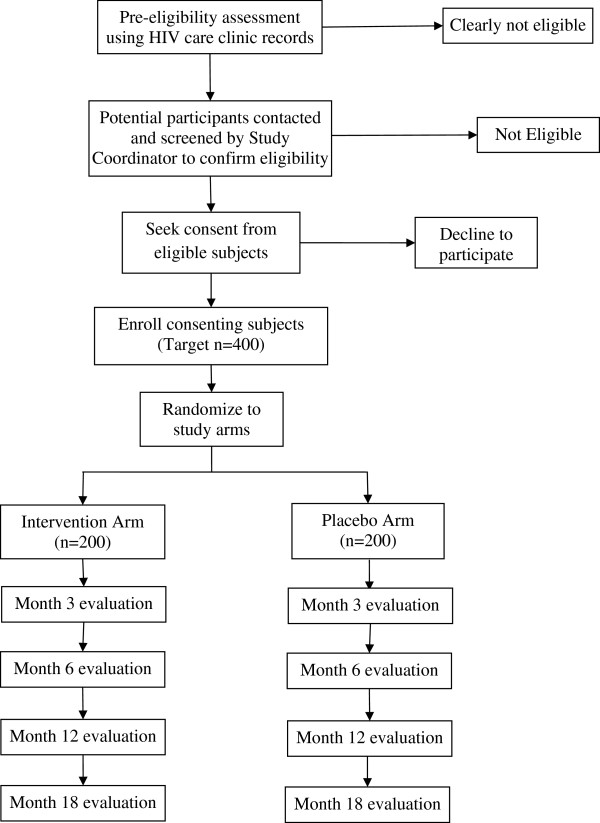
Trial Schema.

Once a patient is deemed eligible and has provided consent, they are randomized to receive the intervention or placebo. Permuted block randomization was used, with a block size of ten. Prior to initiation of the trial, a staff at Harvard School of Public Health (HSPH) not associated with implementation of the trial generated serial numbers from 1 through 400, and randomly assigned these to A and B to indicate allocation to the intervention or placebo groups using SAS version 9.0. Study regimen bottles (manufactured on special order from Tishcon Corp, Salisbury, MD), were then labeled with the serial numbers only, but according to whether the specific serial number was assigned to arm “A” or arm “B”. The study regimen bottles were shipped to Uganda, but the list showing the study arm to which each serial number was assigned remains anonymous and is kept at HSPH and not accessible to study staff in Uganda. At the time of participant enrollment, the Study Pharmacist dispenses the assigned regimen bottles to the participant in sequential order of enrollment. Participants are followed for up to 18 months with evaluations at Baseline, Months 3, 6, 12 and 18. The trial schema is illustrated in Figure 1.

At all times during this trial, participants continue to receive standard medical care according to Uganda’s Ministry of Health (MoH) guidelines. The clinical study staff provides the intervention regimen on a monthly basis to participants as part of their routine medical care. Home visits are performed for individuals who miss their scheduled research study visits. An interim history is conducted to assess participants’ general health and well-being at baseline, months 3, 6, 12 and 18. At the end of each clinic visit, participants receive the trial regimen, instructions on the regimen use, and a follow-up appointment. Table 1 shows the schedule of study events for a typical patient.

**Table 1:** Schedule of events

**ACTIVITY**	**−30 to −7 Days**	**BASELINE**	**M1**	**M2**	**M3**	**M4**	**M5**	**M6**	**M7**	**M8**	**M9**	**M10**	**M11**	**M12**	**M13**	**M14**	**M15**	**M16**	**M17**	**M18**
**I. CASE REPORT FORMS**																				
Pre-Eligibility Assessment	**X**																			
Eligibility Confirmation & Informed Consent		**X**																		
Enrollment & Randomization		**X**																		
Contact Information		**X**																		
Drug Regimen Refill of @ monthly clinic visits		**X**	**X**	**X**	**X**	**X**	**X**	**X**	**X**	**X**	**X**	**X**	**X**	**X**	**X**	**X**	**X**	**X**	**X**	**X**
Background Questionnaire		**X**																		
Health Assessment Questionnaire		**X**			**X**			**X**						**X**						**X**
FFQ		**X**						**X**						**X**						**X**
QoL Questionnaire		**X**						**X**						**X**						**X**
Weight		**X**						**X**						**X**						**X**
**II. LAB TESTS**																				
CD4		**X**			**X**			**X**						**X**						**X**
CBC		**X**						**X**						**X**						**X**
Malaria Parasitemia		**X**						**X**						**X**						**X**
Helminth Infections		**X**						**X**						**X**						**X**
ALT		**X**						**X**						**X**						**X**
Urine Dipstick		**X**						**X**						**X**						**X**
**III. SAMPLES FOR STORAGE**																				
Lipid profile (HDL, LDL, Triglyceride)		**X**						**X**						**X**						**X**
Sample storage for cytokine Elisa (IL2, IL4, IL5, IL6, IL10, IL12, TNF-a, IFN-γ)		**X**						**X**						**X**						**X**
Sample storage for Hep B, Hep C, D4T & Vitamin D levels		**X**						**X**						**X**						**X**

### Measurements

At baseline only, we administer a detailed background information questionnaire to obtain socio-demographic data, including age, parity, education, residence, employment status, socio-economic status variables, marital status, and past/current use of tobacco, alcohol, and other substances.

#### Clinical parameters

Patients undergo a full clinical examination and laboratory parameters including assessments of complete blood counts (CBCs), CD4 counts, and assessments for syphilis, malaria, intestinal parasites. Viral load is not routinely conducted per Uganda’s standard of care. Clinical information is recorded including: (1) a checklist of symptoms – including fever, cough, diarrhea, and anxiety or depression – in the previous month; (2) an assessment of treatment compliance based on self-report and pill count; and (3) anthropometric measurements. In addition, study medical officers perform a complete clinical examination. They code diagnoses according to pre-defined criteria, record all diagnostic tests performed, and document any medications prescribed (e.g. antibiotics, anti-malarials, or changes in HIV treatment). They also document whether participants have attended additional clinic visits outside of their scheduled appointments, record reasons and diagnoses related to these visits, as well as any hospitalizations that occur. We assess quality of life of participants at baseline and every six months thereafter, using the validated, culturally-adapted local language version of the Medical Outcomes Survey-HIV [14].

In cases where patients miss their monthly appointments, a study nurse home visitor visits them at home and encourages them to visit the clinic if their condition permits. For participants who travel out of the study area, we maintain contact with relatives and neighbors in the area of residence to continue monitoring their health status. Although accurate cause-of-death determinations are difficult (post-mortem examination is rarely done), we review hospital records to identify events at or near a patient’s time of death for those who die in the hospital. For those who die elsewhere, we administer a verbal autopsy using a standard form, which includes open- and closed-ended questions to assess clinical parameters and treatments given near the time of death. We will establish cause of death based on consensus decision of two senior HIV clinicians at IDI using all data available.

#### Nutritional assessment

We obtain anthropometric and dietary information to determine nutritional status at each study visit. Weight and mid upper arm circumference (MUAC) are assessed at baseline, month 3 and subsequently six monthly; whereas height is measured at baseline only. Weight and height will be used to calculate body mass index, which together with MUAC will be used to assess nutritional status. We calibrate instruments at regular intervals; and trained study nurses perform anthropometric measurements according to standard techniques, which are standardized at baseline and at regular intervals thereafter.

#### Dietary intake

At baseline and every six months, trained research assistants administer a food frequency questionnaire (FFQs); 24-hour and 7-day diet recalls are conducted at all scheduled study visits. Based on our ongoing trials of nutrition and infection in Tanzania, we are experienced in performing both FFQs and 24-hour recalls in similar HIV-infected populations. In particular, we have spent considerable time in developing an adult FFQ in Tanzania, and we adapted this questionnaire to this study population in Uganda. This FFQ is comprised of an 87-item food recipe list, and each participant is asked to report the frequency and amount of their ‘typical’ consumption for each food item over the previous month. In order to standardize the food quantities consumed, we used commonly used household utensils in Uganda as the portion sizes, to help participants estimate the quantities of foods consumed. If a participant reported to have eaten any food recipe listed, they were asked to indicate the portion sizes consumed using the commonly used household utensils. In a separate exercise, each of the food recipe portion size was weighted, and data on the weight (in grams) of each portion size of each food recipe listed on the FFQ has been stored in a database. During analysis, for each participant, the amount of each item consumed will be multiplied by its nutrient content (derived from nutrient composition tables) and summed over all food items to obtain total intakes for each type of nutrient.

#### Laboratory investigations

A blood specimen is taken from each participant at enrolment and every six months thereafter to measure CD4 counts, CBCs - including hemoglobin levels, total and differential white blood cell counts, hematocrit, bilirubin, creatinin and ALT as part of clinical standard of care for all patients. Outside of standard of care measurements, one repeated measurement of CD4 cell count is conducted at Month 3 to allow us to investigate any acute changes in CD4 cell count in the participants following randomization.

Testing for malaria parasitemia is assessed for all participants every six months regardless of symptoms, and as needed based on clinical indication. In addition, we assess the presence of malaria parasites in the blood for all participants through microscopic investigation of blood smears at baseline, 6, 12 and 18 months. These samples are microscopically analyzed by trained technicians at the IDI laboratory.

In addition to the above, levels of the following lipids: high density lipoproteins (HDL), low density lipoproteins (LDL) and triglycerides will be assessed on the stored serum specimens from 6, 12, and 18 month time points. Vitamin B12 levels will also be assessed on stored specimens on 15% of participants (n=60) to monitor compliance at baseline and at six- and eighteen-month follow-up. We will assess vitamin B12 using a competitive magnetic separation assay on the Technico Immuno-1 analyzer (Bayer, Tarytown, NY); this assay has a day-to-day variability of less than 4.0% for concentrations of between 643 and 852 pg/mL. We also store excess serum specimens from each blood draw at −70 degrees centigrade, and will use them in ancillary studies to examine mechanisms relating to multivitamins and additional outcomes, such as markers of oxidative stress, cytokine proliferation, hepatitis, d4t and serum vitamin levels that may be associated with HIV disease progression.

### Study outcomes

The primary outcomes in this trial are: (1) immune reconstitution, defined as change in CD4 cell count; (2) weight gain; and (3) change in quality of life, defined as overall score on the Medical Outcomes Survey-HIV (MOS-HIV) [14], and individual scores for general health perceptions, physical functioning, pain, energy, role functioning, social functioning, and mental health.

The secondary outcomes include: (1) development of a new or recurrent disease progression event, including all-cause death; (2) changes in antiretroviral treatment defined as switching from first- to second-line therapy; and (3) occurrence of adverse events, including peripheral neuropathy, severe anemia, and diarrhea.

### Statistical issues

#### Statistical power

We calculated powers for testing the efficacy of multivitamins in relation to study outcomes over the 18-month study period, with a sample size of 400, 200 in each study arm, and Type I error rate α=0.05. For continuous endpoints, we calculated power for two sample T test based on the following equation (the example given here is change in CD4 count):

$$\;\text{Power=}\Phi \left\{-{\text{Z}}_{1-\alpha /2}+\left(\surd \text{n}\delta \right)/\left(\sigma_{d}\surd 2\right)\right\}$$

where δ=|μ_1_-μ_2_| and μ_1_ and μ_2_ are the underlying changes in CD4 count in the two treatment arms, σ_d_^2^ = σ_1_^2^+σ_2_^2^-2ρσ_1_σ_2_, σ_1_^2^ and σ_2_^2^ are the variances of baseline and follow-up CD4 counts within a particular treatment arm, ρ is the correlation between baseline and follow-up CD4 counts within a treatment arm over time t, n = (400/2)*(1-F) and F is the proportion of participants lost to follow-up over the study period, and Ф is the cumulative distribution function of the standard normal distribution.

For our calculations, we assumed the null hypothesis that σ_1_^2^, σ_2_^2^, σ_d_^2^ and ρ were the same in the multivitamin and placebo groups; we also assumed that the variance of CD4 counts was the same at baseline and follow-up. We calculated power over a range of assumptions for δ, σ_d_, ρ, and loss to follow-up during 18 months.

For the difference in CD4 count change over the study period between treatment groups, we assumed a change, δ = 40, 50, 60, and 70 cells/mm^3^ based on reported effects of multivitamin supplements in an intervention trial of patients on HAART for at least three months [12]. For the standard deviation of CD4 counts at baseline and follow-up, we assumed σ_1_ = σ_2_ = 175 and 225cells/mm^3^ based on reported values for several trial populations receiving HAART in Brazil and the United States [12,15]. We assumed correlations between baseline and follow-up values of CD4 counts based on the dataset available from our Tanzanian intervention trial [5]; ρ = 0.5, 0.6, and 0.7. For lost to follow-up (F), we used a proportion of 0.10 based on our experience in rigorously conducted randomized trials in Tanzania and Uganda. Table 2 shows estimated power across these various assumptions. There is adequate power to examine the effect of the interventions on immune re-constitution. For example, estimated power assuming δ = 40, σ_1_ = σ_2_ = 175, and ρ = 0.7 was 80%; as expected, the estimated power was higher with bigger mean difference in CD4 change (Table 2).

**Table 2:** Estimated power to detect multivitamin effects on CD4 count change over 18 months of follow-up (n=400)

**Mean difference in CD4 change (δ, in cells/mm**^**3**^**)**	**Standard deviation of CD4 counts at baseline and follow-up (σ**_**1**_**= σ**_**2**_**, in cells/mm**^**3**^**)**		**Correlation of CD4 counts between baseline and follow-up (ρ)**	
		**0.5**	**0.6**	**0.7**
40	175	58	68	80
	225	39	47	59
50	175	77	86	94
	225	56	66	78
60	175	90	95	99
	225	72	81	90
70	175	97	99	>99
	225	84	91	97

Similarly, we calculated power estimates for multivitamin effects on weight change over the study period (Table 3). Assumptions for the mean difference in weight change between treatment and placebo groups (δ=2, 2.5, 3, and 3.5), as well as standard deviations at baseline and follow-up, are based on studies of HAART initiation that reported weight gain over one year in several African countries [16-18]. Although reported values are somewhat variable (ranging from 3 to 9kg), we believe our assumptions are conservative, representing a range of values that are generally smaller than those reported for HAART-related weight gain.

**Table 3:** Estimated power to detect multivitamin effects on weight change over 18 months of follow-up (n=400)

**Mean difference in weight change (δ, in kg)**	**Standard deviation of weight at baseline and follow-up (σ**_**1**_**= σ**_**2**_**, in cells/mm**^**3**^**)**		**Correlation of weight between baseline and follow-up (ρ)**	
		**0.5**	**0.6**	**0.7**
2	9	56	66	78
	11	41	49	61
2.5	9	75	88	96
	11	58	74	86
3	9	88	94	98
	11	74	82	92
3.5	9	96	98	>99
	11	86	92	97

We also calculated power for detecting quality of life changes in the proposed MOS-HIV mental health summary score. Based on a recent study of quality of life in rural Uganda [14], we evaluated a range of parameter estimates: δ = 4, 6, and 8 points and σ_1_ = σ_2_ = 7 and 11; again, we assumed ρ = 0.5, 0.6, and 0.7. This range of score differences assumes less than half of the score improvement associated with HAART over one year of treatment in the Ugandan study (mean difference=+14.2 points on the same mental health summary score) [19]. Furthermore, we assume a 10-20% increase in scores from those reported at baseline in Uganda, which is well within the range of multivitamin effects observed in our Tanzanian trials [5]. Overall, power was estimated at >90% for our proposed study across the range of assumptions we investigated; most estimates were >99%.

#### Planned statistical analysis

For the primary outcomes, we will use intention-to-treat analyses for all primary outcomes. We will employ mixed models to evaluate changes over time in CD4 count, weight gain, and quality of life in relation to assigned treatment group. These models accommodate repeated measures of the outcome, and account for baseline levels as well as concomitant risk factors for the outcome [20].

To evaluate the occurrence of disease progression or mortality, we will use the chi-squared test to compare the proportion of participants who experience these outcomes in the two treatment groups. To assess whether treatment prolongs the time for attaining this endpoint, the log-rank test will be used and Kaplan-Meier curves will be plotted for each treatment group. For adverse drug outcomes (i.e. occurrence of peripheral neuropathy, severe anemia, and diarrhea), we must account for the possibility that participants may experience multiple episodes over the study period. Thus, we will use log-binomial models for repeated outcomes to examine the treatment effect on these outcomes.

### Ethical approval

The trial protocol was approved by the Scientific Review Committee of the Infectious Diseases Institute at Makerere University College of Health Sciences; and the Institutional Review Boards of Harvard School of Public Health and that of Makerere University School of Public Health.

## Discussion

The provision of HAART has significantly decreased HIV-associated morbidity and mortality over the last fifteen years, but despite the substantial benefits of HAART, immune reconstitution may not be complete and the risk of opportunistic infection and death can be high in less developed countries, especially in the first few following treatment initiation. Multivitamins are key factors in maintaining immune function and neutralizing oxidative stress, and among individuals not receiving HAART, but sufficient research data are lacking on whether micronutrient supplementation during HAART has an impact on treatments outcomes [21]. This is especially important on the potential benefits of this affordable adjunct therapy in developing country settings. Since the widespread introduction of HAART, only a few, mainly small, intervention trials have explored multivitamin supplementation in relation to HIV progression. Two non-randomized studies of antioxidant vitamins showed no effects on plasma viral load, although one noted a significant increase in glutathione and glutathione peroxidase [8] and the other identified more pronounced metabolic disturbances in patients with lipoatrophy and hyperlactemia [7]. In a randomized trial of daily vitamin E supplementation [9,10], CD4 counts, the ratio of CD4 to CD8 cells, and plasma viral load remained similar after six months in the treatment versus placebo groups, but supplementation was associated with increased lymphocyte viability. In another trial of daily vitamin A, C, and E supplementation, the supplementation group had significantly elevated concentrations of catalase and superoxide dismutase compared to placebo; still, increases in CD4 count were non-significant in the supplementation group [11]. In a small randomized trial (n=40) of multivitamin supplements given twice daily for three months, those who received supplements had significantly higher absolute CD4 counts and greater mean increases in CD4 count from baseline (p=0.01) [12]. Another small trial (n=30) found that multivitamins (including vitamin E) improved asymptomatic stable chronic hyperlactatemia in HIV-infected patients on long-term HAART [13].

A recently completed trial from Tanzania compared the effects of high doses of supplements including vitamin B complex, vitamin C, and vitamin E with standard doses at the recommended dietary allowance level [22]. High-dose supplementation had no effect on several key measures that reveal HIV disease progression—CD4 count, plasma viral load, body mass index, or hemoglobin level concentration—and did not reduce death or disease progression risks for HIV-infected patients. This study does not rule out the possibility that the high- and standard-dose regimens are equally efficacious. Placebo control trials, such as this ongoing trial in Uganda, are necessary to examine the overall benefit of supplements compared to standard of care, which does not include micronutrient supplements to patients on HAART in many settings. Our trial provides an opportunity to evaluate the safety and efficacy of a single RDA of micronutrients in improving clinical and immunological outcomes, and quality of life among HIV-infected adults receiving HAART in a developing country setting.

## Competing interests

The authors declare that there is no conflict of interest.

## Authors’ contribution

All authors provided comment, read and approved the final version of the manuscript. Study Conceptualization: WF, DG, YM, AE, DB, HW, FWM.

## Pre-publication history

The pre-publication history for this paper can be accessed here:

http://www.biomedcentral.com/1471-2334/12/304/prepub
